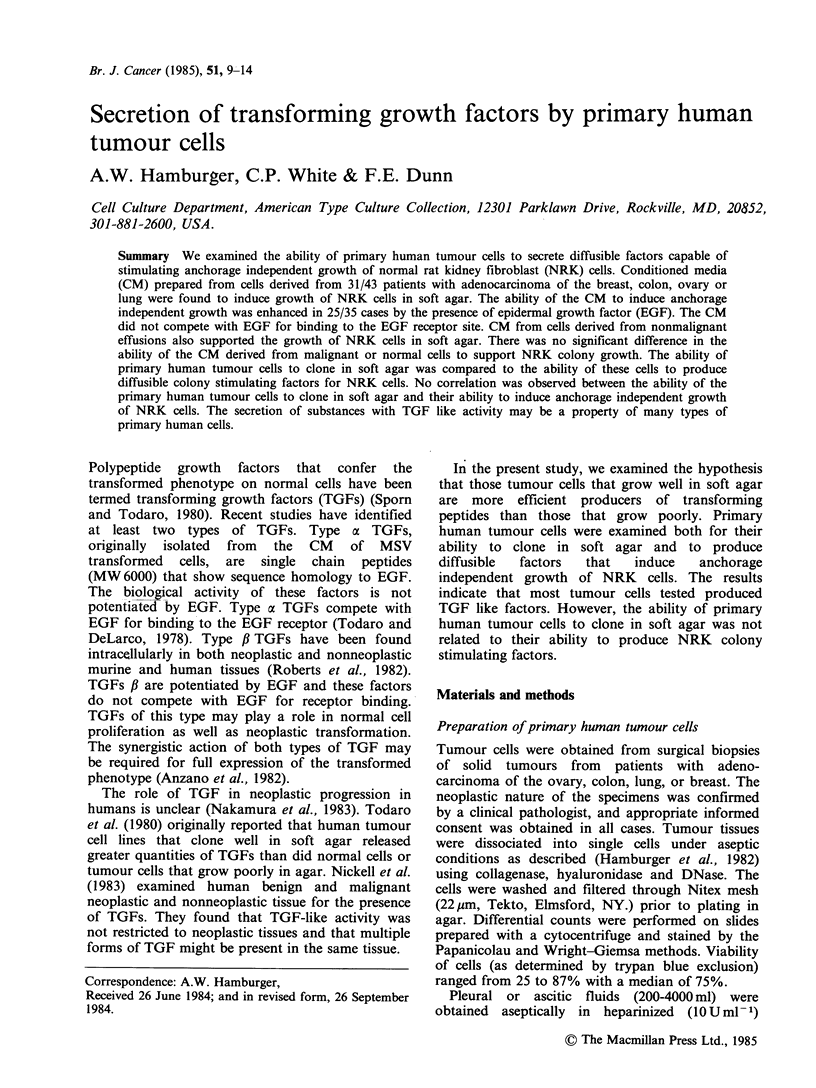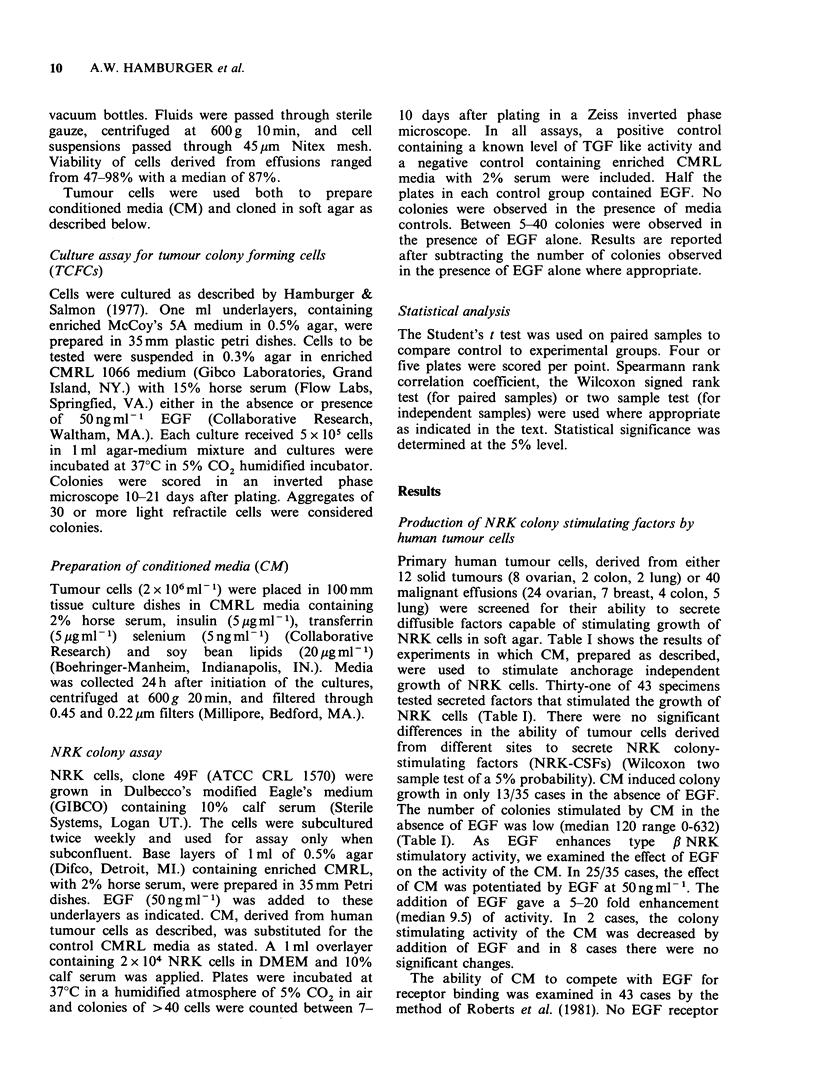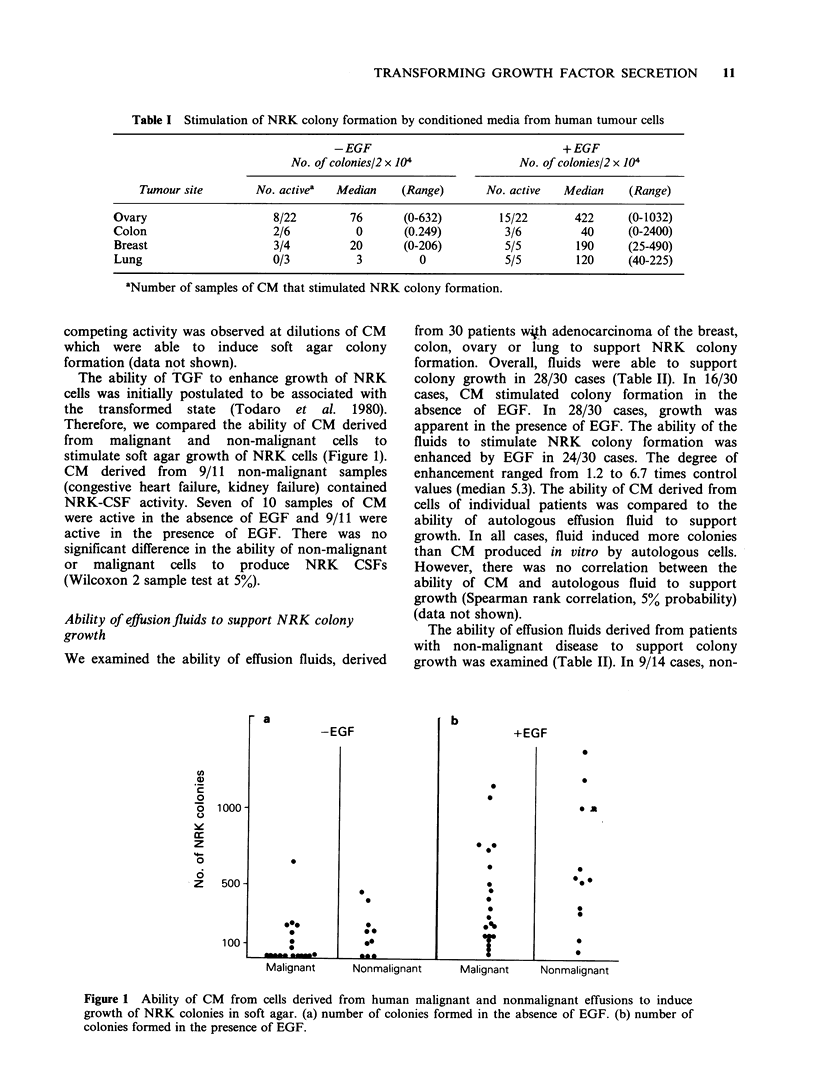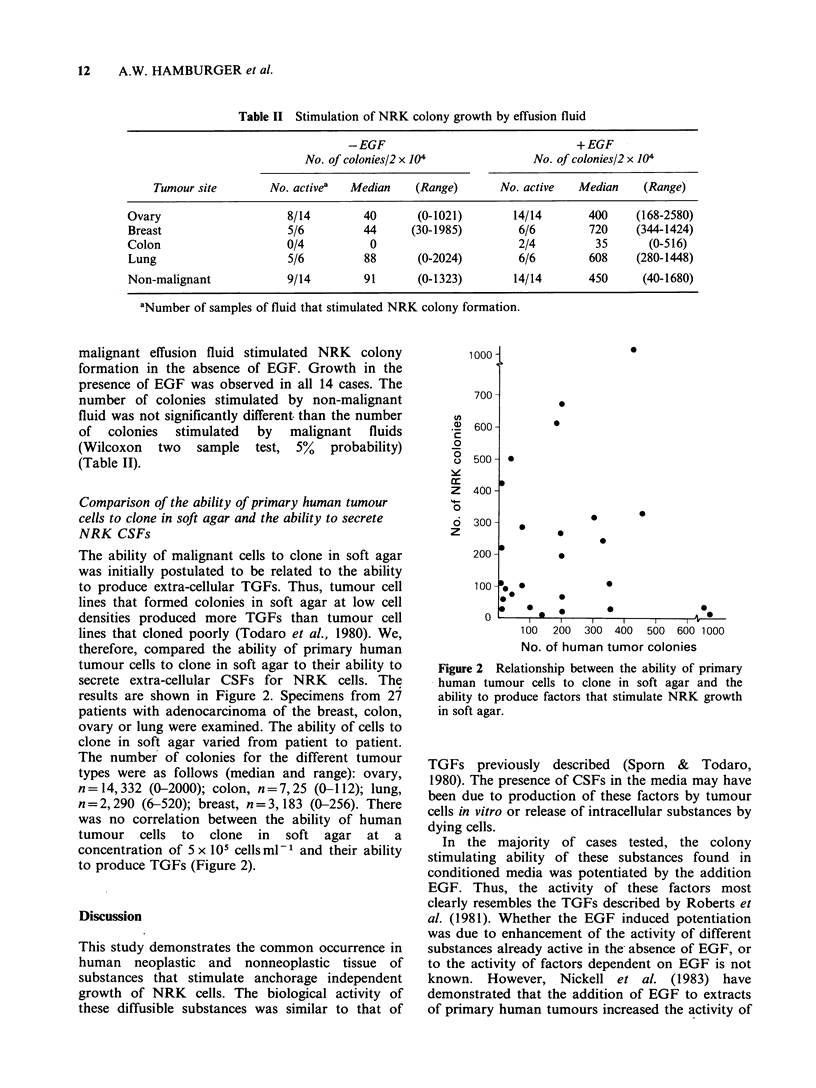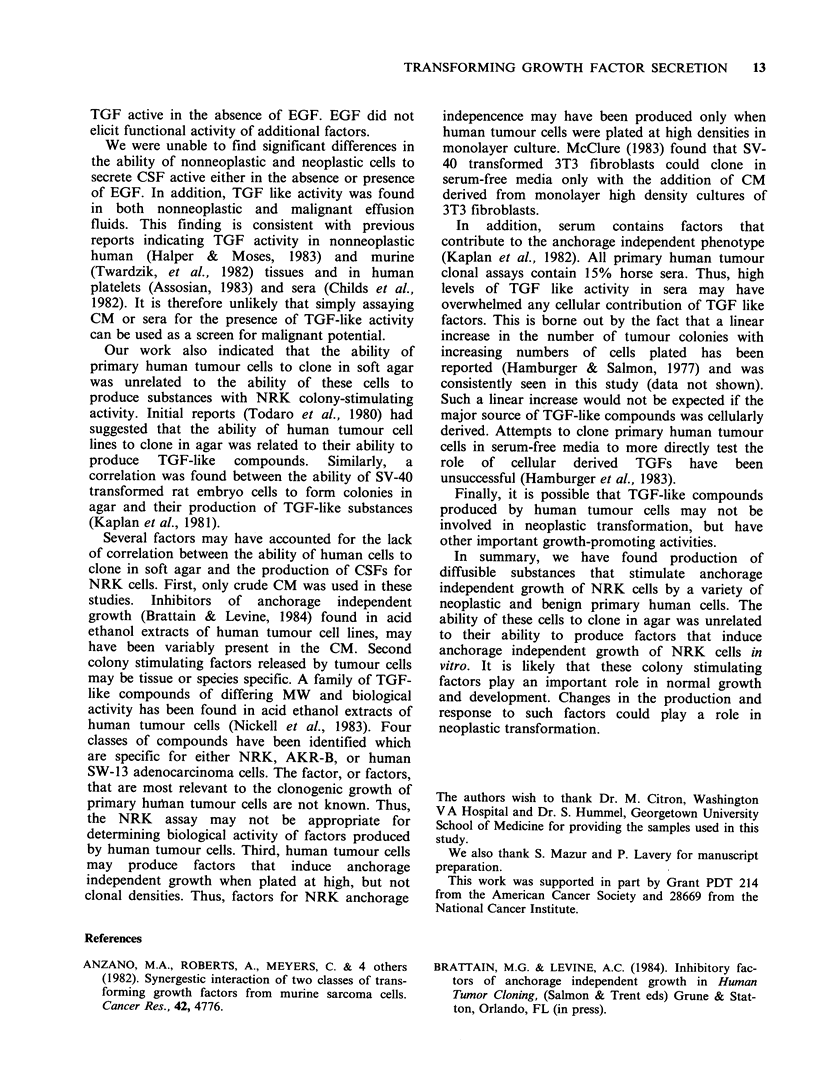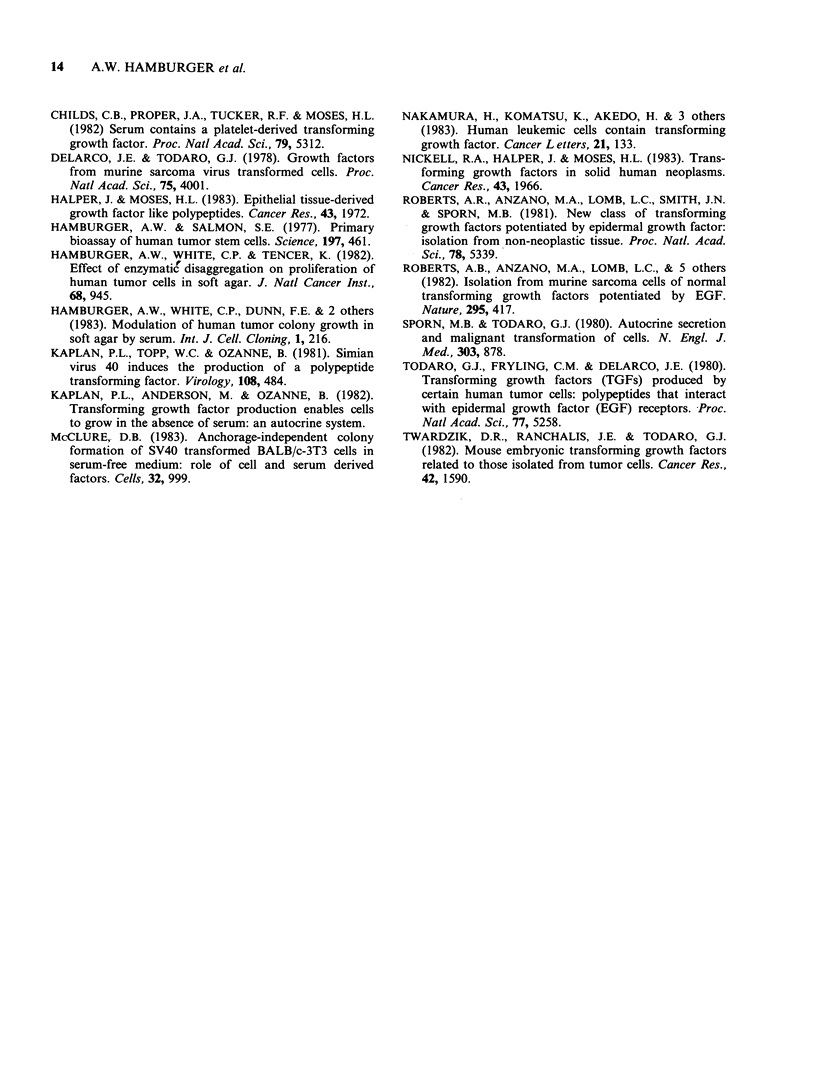# Secretion of transforming growth factors by primary human tumour cells.

**DOI:** 10.1038/bjc.1985.2

**Published:** 1985-01

**Authors:** A. W. Hamburger, C. P. White, F. E. Dunn

## Abstract

We examined the ability of primary human tumour cells to secrete diffusible factors capable of stimulating anchorage independent growth of normal rat kidney fibroblast (NRK) cells. Conditioned media (CM) prepared from cells derived from 31/43 patients with adenocarcinoma of the breast, colon, ovary or lung were found to induce growth of NRK cells in soft agar. The ability of the CM to induce anchorage independent growth was enhanced in 25/35 cases by the presence of epidermal growth factor (EGF). The CM did not compete with EGF for binding to the EGF receptor site. CM from cells derived from nonmalignant effusions also supported the growth of NRK cells in soft agar. There was no significant difference in the ability of the CM derived from malignant or normal cells to support NRK colony growth. The ability of primary human tumour cells to clone in soft agar was compared to the ability of these cells to produce diffusible colony stimulating factors for NRK cells. No correlation was observed between the ability of the primary human tumour cells to clone in soft agar and their ability to induce anchorage independent growth of NRK cells. The secretion of substances with TGF like activity may be a property of many types of primary human cells.


*Br. J. Cancer* (1985), **51**, 9–14

### Secretion of transforming growth factors by primary human tumour cells

A.W. Hamburger, C.P. White & F.E. Dunn

*Cell Culture Department, American Type Culture Collection, 12301 Parklawn Drive, Rockville, MD, 20852, 301-881-2600, USA.*

**Summary** We examined the ability of primary human tumour cells to secrete diffusible factors capable of stimulating anchorage independent growth of normal rat kidney fibroblast (NRK) cells. Conditioned media (CM) prepared from cells derived from 31/43 patients with adenocarcinoma of the breast, colon, ovary or lung were found to induce growth of NRK cells in soft agar. The ability of the CM to induce anchorage independent growth was enhanced in 25/35 cases by the presence of epidermal growth factor (EGF). The CM did not compete with EGF for binding to the EGF receptor site. CM from cells derived from nonmalignant effusions also supported the growth of NRK cells in soft agar. There was no significant difference in the ability of the CM derived from malignant or normal cells to support NRK colony growth. The ability of primary human tumour cells to clone in soft agar was compared to the ability of these cells to produce diffusible colony stimulating factors for NRK cells. No correlation was observed between the ability of the primary human tumour cells to clone in soft agar and their ability to induce anchorage independent growth of NRK cells. The secretion of substances with TGF like activity may be a property of many types of primary human cells.

Polypeptide growth factors that confer the transformed phenotype on normal cells have been termed transforming growth factors (TGFs) (Sporn and Todaro, 1980). Recent studies have identified at least two types of TGFs. Type α TGFs, originally isolated from the CM of MSV transformed cells, are single chain peptides (MW 6000) that show sequence homology to EGF. The biological activity of these factors is not potentiated by EGF. Type α TGFs compete with EGF for binding to the EGF receptor (Todaro and DeLarco, 1978). Type β TGFs have been found intracellularly in both neoplastic and nonneoplastic murine and human tissues (Roberts *et al.*, 1982). TGFs β are potentiated by EGF and these factors do not compete with EGF for receptor binding. TGFs of this type may play a role in normal cell proliferation as well as neoplastic transformation. The synergistic action of both types of TGF may be required for full expression of the transformed phenotype (Anzano *et al.*, 1982).

The role of TGF in neoplastic progression in humans is unclear (Nakamura *et al.*, 1983). Todaro *et al.* (1980) originally reported that human tumour cell lines that clone well in soft agar released greater quantities of TGFs than did normal cells or tumour cells that grow poorly in agar. Nickell *et al.* (1983) examined human benign and malignant neoplastic and nonneoplastic tissue for the presence of TGFs. They found that TGF-like activity was not restricted to neoplastic tissues and that multiple forms of TGF might be present in the same tissue.

Correspondence: A.W. Hamburger,
Received 26 June 1984; and in revised form, 26 September 1984.

In the present study, we examined the hypothesis that those tumour cells that grow well in soft agar are more efficient producers of transforming peptides than those that grow poorly. Primary human tumour cells were examined both for their ability to clone in soft agar and to produce diffusible factors that induce anchorage independent growth of NRK cells. The results indicate that most tumour cells tested produced TGF like factors. However, the ability of primary human tumour cells to clone in soft agar was not related to their ability to produce NRK colony stimulating factors.

### Materials and methods

### *Preparation of primary human tumour cells*

Tumour cells were obtained from surgical biopsies of solid tumours from patients with adenocarcinoma of the ovary, colon, lung, or breast. The neoplastic nature of the specimens was confirmed by a clinical pathologist, and appropriate informed consent was obtained in all cases. Tumour tissues were dissociated into single cells under aseptic conditions as described (Hamburger *et al.*, 1982) using collagenase, hyaluronidase and DNase. The cells were washed and filtered through Nitex mesh (22 μm, Tekto, Elmsford, NY.) prior to plating in agar. Differential counts were performed on slides prepared with a cytocentrifuge and stained by the Papanicolau and Wright–Giemsa methods. Viability of cells (as determined by trypan blue exclusion) ranged from 25 to 87% with a median of 75%.

Pleural or ascitic fluids (200-4000 ml) were obtained aseptically in heparinized ($10\,U\,ml^{-1}$)

© The Macmillan Press Ltd., 1985

vacuum bottles. Fluids were passed through sterile gauze, centrifuged at 600 g 10 min, and cell suspensions passed through 45 μm Nitex mesh. Viability of cells derived from effusions ranged from 47–98% with a median of 87%.

Tumour cells were used both to prepare conditioned media (CM) and cloned in soft agar as described below.

*Culture assay for tumour colony forming cells (TCFCs)*

Cells were cultured as described by Hamburger & Salmon (1977). One ml underlayers, containing enriched McCoy's 5A medium in 0.5% agar, were prepared in 35 mm plastic petri dishes. Cells to be tested were suspended in 0.3% agar in enriched CMRL 1066 medium (Gibco Laboratories, Grand Island, NY.) with 15% horse serum (Flow Labs, Springfied, VA.) either in the absence or presence of 50 ng ml$^{-1}$ EGF (Collaborative Research, Waltham, MA.). Each culture received $5 \times 10^5$ cells in 1 ml agar-medium mixture and cultures were incubated at 37°C in 5% $CO_2$ humidified incubator. Colonies were scored in an inverted phase microscope 10–21 days after plating. Aggregates of 30 or more light refractile cells were considered colonies.

*Preparation of conditioned media (CM)*

Tumour cells ($2 \times 10^6$ ml$^{-1}$) were placed in 100 mm tissue culture dishes in CMRL media containing 2% horse serum, insulin (5 μg ml$^{-1}$), transferrin (5 μg ml$^{-1}$) selenium (5 ng ml$^{-1}$) (Collaborative Research) and soy bean lipids (20 μg ml$^{-1}$) (Boehringer-Manheim, Indianapolis, IN.). Media was collected 24 h after initiation of the cultures, centrifuged at 600 *g* 20 min, and filtered through 0.45 and 0.22 μm filters (Millipore, Bedford, MA.).

*NRK colony assay*

NRK cells, clone 49F (ATCC CRL 1570) were grown in Dulbecco's modified Eagle's medium (GIBCO) containing 10% calf serum (Sterile Systems, Logan UT.). The cells were subcultured twice weekly and used for assay only when subconfluent. Base layers of 1 ml of 0.5% agar (Difco, Detroit, MI.) containing enriched CMRL, with 2% horse serum, were prepared in 35 mm Petri dishes. EGF (50 ng ml$^{-1}$) was added to these underlayers as indicated. CM, derived from human tumour cells as described, was substituted for the control CMRL media as stated. A 1 ml overlayer containing $2 \times 10^4$ NRK cells in DMEM and 10% calf serum was applied. Plates were incubated at 37°C in a humidified atmosphere of 5% $CO_2$ in air and colonies of >40 cells were counted between 7–10 days after plating in a Zeiss inverted phase microscope. In all assays, a positive control containing a known level of TGF like activity and a negative control containing enriched CMRL media with 2% serum were included. Half the plates in each control group contained EGF. No colonies were observed in the presence of media controls. Between 5–40 colonies were observed in the presence of EGF alone. Results are reported after subtracting the number of colonies observed in the presence of EGF alone where appropriate.

*Statistical analysis*

The Student's *t* test was used on paired samples to compare control to experimental groups. Four or five plates were scored per point. Spearmann rank correlation coefficient, the Wilcoxon signed rank test (for paired samples) or two sample test (for independent samples) were used where appropriate as indicated in the text. Statistical significance was determined at the 5% level.

### Results

*Production of NRK colony stimulating factors by human tumour cells*

Primary human tumour cells, derived from either 12 solid tumours (8 ovarian, 2 colon, 2 lung) or 40 malignant effusions (24 ovarian, 7 breast, 4 colon, 5 lung) were screened for their ability to secrete diffusible factors capable of stimulating growth of NRK cells in soft agar. Table I shows the results of experiments in which CM, prepared as described, were used to stimulate anchorage independent growth of NRK cells. Thirty-one of 43 specimens tested secreted factors that stimulated the growth of NRK cells (Table I). There were no significant differences in the ability of tumour cells derived from different sites to secrete NRK colony-stimulating factors (NRK-CSFs) (Wilcoxon two sample test of a 5% probability). CM induced colony growth in only 13/35 cases in the absence of EGF. The number of colonies stimulated by CM in the absence of EGF was low (median 120 range 0-632) (Table I). As EGF enhances type β NRK stimulatory activity, we examined the effect of EGF on the activity of the CM. In 25/35 cases, the effect of CM was potentiated by EGF at 50 ng ml$^{-1}$. The addition of EGF gave a 5–20 fold enhancement (median 9.5) of activity. In 2 cases, the colony stimulating activity of the CM was decreased by addition of EGF and in 8 cases there were no significant changes.

The ability of CM to compete with EGF for receptor binding was examined in 43 cases by the method of Roberts *et al.* (1981). No EGF receptor

**Table I** Stimulation of NRK colony formation by conditioned media from human tumour cells

| | *−EGF No. of colonies/2 × 10⁴* | | | *+EGF No. of colonies/2 × 10⁴* | | |
|---|---|---|---|---|---|---|
| *Tumour site* | *No. active*[a] | *Median* | *(Range)* | *No. active* | *Median* | *(Range)* |
| Ovary | 8/22 | 76 | (0-632) | 15/22 | 422 | (0-1032) |
| Colon | 2/6 | 0 | (0.249) | 3/6 | 40 | (0-2400) |
| Breast | 3/4 | 20 | (0-206) | 5/5 | 190 | (25-490) |
| Lung | 0/3 | 3 | 0 | 5/5 | 120 | (40-225) |

[a]Number of samples of CM that stimulated NRK colony formation.

competing activity was observed at dilutions of CM which were able to induce soft agar colony formation (data not shown).

The ability of TGF to enhance growth of NRK cells was initially postulated to be associated with the transformed state (Todaro *et al.* 1980). Therefore, we compared the ability of CM derived from malignant and non-malignant cells to stimulate soft agar growth of NRK cells (Figure 1). CM derived from 9/11 non-malignant samples (congestive heart failure, kidney failure) contained NRK-CSF activity. Seven of 10 samples of CM were active in the absence of EGF and 9/11 were active in the presence of EGF. There was no significant difference in the ability of non-malignant or malignant cells to produce NRK CSFs (Wilcoxon 2 sample test at 5%).

*Ability of effusion fluids to support NRK colony growth*

We examined the ability of effusion fluids, derived from 30 patients with adenocarcinoma of the breast, colon, ovary or lung to support NRK colony formation. Overall, fluids were able to support colony growth in 28/30 cases (Table II). In 16/30 cases, CM stimulated colony formation in the absence of EGF. In 28/30 cases, growth was apparent in the presence of EGF. The ability of the fluids to stimulate NRK colony formation was enhanced by EGF in 24/30 cases. The degree of enhancement ranged from 1.2 to 6.7 times control values (median 5.3). The ability of CM derived from cells of individual patients was compared to the ability of autologous effusion fluid to support growth. In all cases, fluid induced more colonies than CM produced *in vitro* by autologous cells. However, there was no correlation between the ability of CM and autologous fluid to support growth (Spearman rank correlation, 5% probability) (data not shown).

The ability of effusion fluids derived from patients with non-malignant disease to support colony growth was examined (Table II). In 9/14 cases, non-

**Figure 1** Ability of CM from cells derived from human malignant and nonmalignant effusions to induce growth of NRK colonies in soft agar. (a) number of colonies formed in the absence of EGF. (b) number of colonies formed in the presence of EGF.

**Table II** Stimulation of NRK colony growth by effusion fluid

| | −*EGF* No. of colonies/2 × 10⁴ | | | +*EGF* No. of colonies/2 × 10⁴ | | |
|---|---|---|---|---|---|---|
| *Tumour site* | *No. active*[a] | *Median* | (*Range*) | *No. active* | *Median* | (*Range*) |
| Ovary | 8/14 | 40 | (0-1021) | 14/14 | 400 | (168-2580) |
| Breast | 5/6 | 44 | (30-1985) | 6/6 | 720 | (344-1424) |
| Colon | 0/4 | 0 | | 2/4 | 35 | (0-516) |
| Lung | 5/6 | 88 | (0-2024) | 6/6 | 608 | (280-1448) |
| Non-malignant | 9/14 | 91 | (0-1323) | 14/14 | 450 | (40-1680) |

[a]Number of samples of fluid that stimulated NRK colony formation.

malignant effusion fluid stimulated NRK colony formation in the absence of EGF. Growth in the presence of EGF was observed in all 14 cases. The number of colonies stimulated by non-malignant fluid was not significantly different than the number of colonies stimulated by malignant fluids (Wilcoxon two sample test, 5% probability) (Table II).

*Comparison of the ability of primary human tumour cells to clone in soft agar and the ability to secrete NRK CSFs*

The ability of malignant cells to clone in soft agar was initially postulated to be related to the ability to produce extra-cellular TGFs. Thus, tumour cell lines that formed colonies in soft agar at low cell densities produced more TGFs than tumour cell lines that cloned poorly (Todaro *et al.*, 1980). We, therefore, compared the ability of primary human tumour cells to clone in soft agar to their ability to secrete extra-cellular CSFs for NRK cells. The results are shown in Figure 2. Specimens from 27 patients with adenocarcinoma of the breast, colon, ovary or lung were examined. The ability of cells to clone in soft agar varied from patient to patient. The number of colonies for the different tumour types were as follows (median and range): ovary, $n=14, 332$ (0–2000); colon, $n=7, 25$ (0–112); lung, $n=2, 290$ (6–520); breast, $n=3, 183$ (0–256). There was no correlation between the ability of human tumour cells to clone in soft agar at a concentration of $5 \times 10^5$ cells ml$^{-1}$ and their ability to produce TGFs (Figure 2).

**Figure 2** Relationship between the ability of primary human tumour cells to clone in soft agar and the ability to produce factors that stimulate NRK growth in soft agar.

### Discussion

This study demonstrates the common occurrence in human neoplastic and nonneoplastic tissue of substances that stimulate anchorage independent growth of NRK cells. The biological activity of these diffusible substances was similar to that of TGFs previously described (Sporn & Todaro, 1980). The presence of CSFs in the media may have been due to production of these factors by tumour cells *in vitro* or release of intracellular substances by dying cells.

In the majority of cases tested, the colony stimulating ability of these substances found in conditioned media was potentiated by the addition EGF. Thus, the activity of these factors most clearly resembles the TGFs described by Roberts *et al.* (1981). Whether the EGF induced potentiation was due to enhancement of the activity of different substances already active in the absence of EGF, or to the activity of factors dependent on EGF is not known. However, Nickell *et al.* (1983) have demonstrated that the addition of EGF to extracts of primary human tumours increased the activity of

TGF active in the absence of EGF. EGF did not elicit functional activity of additional factors.

We were unable to find significant differences in the ability of nonneoplastic and neoplastic cells to secrete CSF active either in the absence or presence of EGF. In addition, TGF like activity was found in both nonneoplastic and malignant effusion fluids. This finding is consistent with previous reports indicating TGF activity in nonneoplastic human (Halper & Moses, 1983) and murine (Twardzik, *et al.,* 1982) tissues and in human platelets (Assosian, 1983) and sera (Childs *et al.,* 1982). It is therefore unlikely that simply assaying CM or sera for the presence of TGF-like activity can be used as a screen for malignant potential.

Our work also indicated that the ability of primary human tumour cells to clone in soft agar was unrelated to the ability of these cells to produce substances with NRK colony-stimulating activity. Initial reports (Todaro *et al.,* 1980) had suggested that the ability of human tumour cell lines to clone in agar was related to their ability to produce TGF-like compounds. Similarly, a correlation was found between the ability of SV-40 transformed rat embryo cells to form colonies in agar and their production of TGF-like substances (Kaplan *et al.*, 1981).

Several factors may have accounted for the lack of correlation between the ability of human cells to clone in soft agar and the production of CSFs for NRK cells. First, only crude CM was used in these studies. Inhibitors of anchorage independent growth (Brattain & Levine, 1984) found in acid ethanol extracts of human tumour cell lines, may have been variably present in the CM. Second colony stimulating factors released by tumour cells may be tissue or species specific. A family of TGF-like compounds of differing MW and biological activity has been found in acid ethanol extracts of human tumour cells (Nickell *et al.*, 1983). Four classes of compounds have been identified which are specific for either NRK, AKR-B, or human SW-13 adenocarcinoma cells. The factor, or factors, that are most relevant to the clonogenic growth of primary human tumour cells are not known. Thus, the NRK assay may not be appropriate for determining biological activity of factors produced by human tumour cells. Third, human tumour cells may produce factors that induce anchorage independent growth when plated at high, but not clonal densities. Thus, factors for NRK anchorage independence may have been produced only when human tumour cells were plated at high densities in monolayer culture. McClure (1983) found that SV-40 transformed 3T3 fibroblasts could clone in serum-free media only with the addition of CM derived from monolayer high density cultures of 3T3 fibroblasts.

In addition, serum contains factors that contribute to the anchorage independent phenotype (Kaplan *et al.,* 1982). All primary human tumour clonal assays contain 15% horse sera. Thus, high levels of TGF like activity in sera may have overwhelmed any cellular contribution of TGF like factors. This is borne out by the fact that a linear increase in the number of tumour colonies with increasing numbers of cells plated has been reported (Hamburger & Salmon, 1977) and was consistently seen in this study (data not shown). Such a linear increase would not be expected if the major source of TGF-like compounds was cellularly derived. Attempts to clone primary human tumour cells in serum-free media to more directly test the role of cellular derived TGFs have been unsuccessful (Hamburger *et al.,* 1983).

Finally, it is possible that TGF-like compounds produced by human tumour cells may not be involved in neoplastic transformation, but have other important growth-promoting activities.

In summary, we have found production of diffusible substances that stimulate anchorage independent growth of NRK cells by a variety of neoplastic and benign primary human cells. The ability of these cells to clone in agar was unrelated to their ability to produce factors that induce anchorage independent growth of NRK cells *in vitro.* It is likely that these colony stimulating factors play an important role in normal growth and development. Changes in the production and response to such factors could play a role in neoplastic transformation.

The authors wish to thank Dr. M. Citron, Washington VA Hospital and Dr. S. Hummel, Georgetown University School of Medicine for providing the samples used in this study.

We also thank S. Mazur and P. Lavery for manuscript preparation.

This work was supported in part by Grant PDT 214 from the American Cancer Society and 28669 from the National Cancer Institute.

**References**

ANZANO, M.A., ROBERTS, A., MEYERS, C. & 4 others (1982). Synergestic interaction of two classes of transforming growth factors from murine sarcoma cells. *Cancer Res.*, **42,** 4776.

BRATTAIN, M.G. & LEVINE, A.C. (1984). Inhibitory factors of anchorage independent growth in *Human Tumor Cloning,* (Salmon & Trent eds) Grune & Statton, Orlando, FL (in press).

CHILDS, C.B., PROPER, J.A., TUCKER, R.F. & MOSES, H.L. (1982) Serum contains a platelet-derived transforming growth factor. *Proc. Natl Acad. Sci.*, **79**, 5312.

DELARCO, J.E. & TODARO, G.J. (1978). Growth factors from murine sarcoma virus transformed cells. *Proc. Natl Acad. Sci.*, **75**, 4001.

HALPER, J. & MOSES, H.L. (1983). Epithelial tissue-derived growth factor like polypeptides. *Cancer Res.*, **43**, 1972.

HAMBURGER, A.W. & SALMON, S.E. (1977). Primary bioassay of human tumor stem cells. *Science*, **197**, 461.

HAMBURGER, A.W., WHITE, C.P. & TENCER, K. (1982). Effect of enzymatic disaggregation on proliferation of human tumor cells in soft agar. *J. Natl Cancer Inst.*, **68**, 945.

HAMBURGER, A.W., WHITE, C.P., DUNN, F.E. & 2 others (1983). Modulation of human tumor colony growth in soft agar by serum. *Int. J. Cell. Cloning*, **1**, 216.

KAPLAN, P.L., TOPP, W.C. & OZANNE, B. (1981). Simian virus 40 induces the production of a polypeptide transforming factor. *Virology*, **108**, 484.

KAPLAN, P.L., ANDERSON, M. & OZANNE, B. (1982). Transforming growth factor production enables cells to grow in the absence of serum: an autocrine system.

McCLURE, D.B. (1983). Anchorage-independent colony formation of SV40 transformed BALB/c-3T3 cells in serum-free medium: role of cell and serum derived factors. *Cells*, **32**, 999.

NAKAMURA, H., KOMATSU, K., AKEDO, H. & 3 others (1983). Human leukemic cells contain transforming growth factor. *Cancer Letters*, **21**, 133.

NICKELL, R.A., HALPER, J. & MOSES, H.L. (1983). Transforming growth factors in solid human neoplasms. *Cancer Res.*, **43**, 1966.

ROBERTS, A.R., ANZANO, M.A., LOMB, L.C., SMITH, J.N. & SPORN, M.B. (1981). New class of transforming growth factors potentiated by epidermal growth factor: isolation from non-neoplastic tissue. *Proc. Natl. Acad. Sci.*, **78**, 5339.

ROBERTS, A.B., ANZANO, M.A., LOMB, L.C., & 5 others (1982). Isolation from murine sarcoma cells of normal transforming growth factors potentiated by EGF. *Nature*, **295**, 417.

SPORN, M.B. & TODARO, G.J. (1980). Autocrine secretion and malignant transformation of cells. *N. Engl. J. Med.*, **303**, 878.

TODARO, G.J., FRYLING, C.M. & DELARCO, J.E. (1980). Transforming growth factors (TGFs) produced by certain human tumor cells: polypeptides that interact with epidermal growth factor (EGF) receptors. *Proc. Natl Acad. Sci.*, **77**, 5258.

TWARDZIK, D.R., RANCHALIS, J.E. & TODARO, G.J. (1982). Mouse embryonic transforming growth factors related to those isolated from tumor cells. *Cancer Res.*, **42**, 1590.